# Sensory function in the faces of patients with facial palsy: A prospective observational study using quantitative sensory testing

**DOI:** 10.3389/fpain.2022.1041905

**Published:** 2022-12-19

**Authors:** Gerd Fabian Volk, Marianna Döhler, Carsten M. Klinger, Thomas Weiss, Orlando Guntinas-Lichius

**Affiliations:** ^1^ Department of Otorhinolaryngology, Jena University Hospital, Jena, Germany; ^2^ Facial-Nerve-Center Jena, Jena University Hospital, Jena, Germany; ^3^ Center for Rare Diseases, Jena University Hospital, Jena, Germany; ^4^ Department of Neurology, Jena University Hospital, Jena, Germany; ^5^ Department of Psychology, Clinical Psychology, Friedrich Schiller University, Jena, Germany

**Keywords:** facial paralysis, Bell’s palsy, quantitative sensory testing, facial sensibility, trigeminal nerve

## Abstract

**Objectives/Hypothesis:**

To determine the sensory function of both sides of the face in patients with acute or chronic facial palsy.

**Study design:**

Prospective observational study.

**Methods:**

The standardized quantitative sensory testing (QST) protocol of the German Research Network on Neuropathic Pain (DFNS), including thermal or mechanical stimuli (touch, pain, vibration, and pressure), was used to investigate somatosensory function in the faces of patients. A patient-reported outcome measures for the assessment of disturbed facial comfort or facial pain, the facial Clinimetric Evaluation Scale (FaCE) Facial Comfort Subscale, and the 36-Item Short Form Survey (SF-36) pain subdomain were used.

**Results:**

A total of 29 patients (22 female, median age of 48 years; 7 acute palsy; 22 chronic palsy; House-Brackmann grade II–VI) were included. The median FaCE Facial Comfort Subscale score and the median SF-36 pain subdomain score were 50 and 100, respectively. Most patients had, at an individual level, a normal sensory function in all or most tests. On average, the frequencies for all parameters were not different between the paretic side and the contralateral side (all *p* > 0.05). Additionally, when *z*-scores were used to compare our patient sample with healthy controls from the DFNS reference database, there was no difference between the paretic side and the contralateral side (all *p* > 0.05). Furthermore, there were no differences between patients with acute facial palsy and those with chronic facial palsy (all *p* > 0.05). The FaCE Facial Comfort Subscale score and the SF-36 pain subdomain score did not correlate with the QST parameters (all *p* > 0.05).

**Conclusion:**

Patients with acute or chronic unilateral peripheral facial palsy had normal sensory function on the paretic and contralateral sides compared with the reference values of healthy controls, and there was no significant difference between the sides. The numbness frequently felt in the affected hemiface is not related to a peripheral sensory disorder and is most likely a manifestation of an unsolved cortical somatosensory-motor mismatch.

## Introduction

The facial nerve is a pure motor nerve formed by the facial motoneurons, which are mainly directed to the facial muscles (1). Nevertheless, the nerve has three other components. Parasympathetic and sensory nerve fibers from the intermediate nerve partly accompany the facial nerves to their targets, i.e., lacrimal, submandibular, sublingual glands, and anterior two-thirds of the tongue (2). The sensory auricular branch of the facial nerve seems to be more variable, and it is believed to provide sensory innervation to the external auditory canal and pinna (3, 4). The latter might explain why some patients with vestibular schwannoma describe a hypoesthesia in an area of the external auditory canal (Hitselberger's sign), or why some patients with herpes zoster oticus only show a zoster eruption in parts of the external auditory canal or pinna (5, 6). An ipsilateral numbness, mentioned by many patients, is also typical for Bell's palsy in clinical practice (7). A good explanation has not yet been offered. It is speculated that the underlying reason in some Bell's palsy cases is a viral disease with additional affection of the greater petrosal nerve. The greater petrosal nerve, again, has connections to the trigeminal nerve. The viral infection might have spread along these anatomical connections (7). More simply, it is often said, “because it is paretic, it feels different” (7). The hypothesis of somatosensory-motor mismatch puts forward an explanation for this phenomenon (8). With regard to mismatch-based learning, the palsy is caused by a peripheral deafferentation of facial muscles, whereas the somatosensory afference of the face is not affected (8). The inability to move facial muscles combined with an unaffected somatosensory afference causes a mismatch signal between intended and perceived movements (8). It is hypothesized that the decreased functional connectivity at the cortical level reflects an unsuccessful sensorimotor adaptation process due to the inability to solve the somatosensory-motor mismatch (8). This might explain why “because it is paretic, it feels different”.

The prevalence of such different feelings reported by patients with facial palsy is unknown. Standardized clinical studies on sensory function in patients with facial palsy are sparse. The few published studies include only some aspects of somatosensory function in the face (9–11). Examination of somatosensory symptoms should include all afferent nerve fiber classes (A*β*, A*δ*, and C fibers) (12). Generally, the face exhibits lower sensory thresholds than any other body region (13). The German Research Network for Neuropathic Pain has proposed a standardized examination protocol (13), i.e., the protocol for quantitative sensory testing (QST) for all fiber types. An important part of the QST analysis is the *z*-score transformation, as sensitivity thresholds are largely dependent on gender and age (12). Furthermore, by using a 95% confidence interval of the mean reference value, QST offers a clear cutoff for sensory abnormalities and has become an international standard (14). Reference data for several body sites have been published (15, 16). Therefore, this protocol would allow the characterization of somatosensory functions in patients with Bell's palsy (13, 17). Consequently, the aim of the present study was to conduct the first comprehensive examination of somatosensory functions in patients with acute or chronic facial palsy.

## Materials and methods

### Study design and setting

This prospective observational study was performed at the Department of Otorhinolaryngology, Jena University Hospital, Germany. Approval for the study was obtained through the local institutional ethics review board (No. 4289-12/14). Written informed consent was obtained from all study participants. Exclusion criteria were as follows: bilateral facial palsy, central facial palsy, peripheral neuropathy; disease of the trigeminal nerve; history of facial trauma; age <18 years.

### Facial function classification

All patients received a routine and standard electrophysiological assessment (electroneurography, blink reflex testing, and needle electromyography) to confirm the diagnosis of peripheral facial palsy (18, 19). Facial nerve function was graded using the House-Brackmann (HB) facial nerve grading system (20). The Facial Clinimetric Evaluation (FaCE) scale was used as facial-specific patient-reported outcome (21). The FaCE has six independent domains. Only data from the facial comfort domain and the total score are reported here. The FaCE facial comfort domain was chosen as the underlying question asked about discomfort and strange feeling in the face. The 36-item SF-36 Health Survey (SF-36) measured general quality of life (22). Only data from the pain and general health subdomains are reported here. Each FaCE score and SF-36 domain score ranged from 0 (worst) to 100 (best).

### Quantitative sensory testing

**Figure 1:**
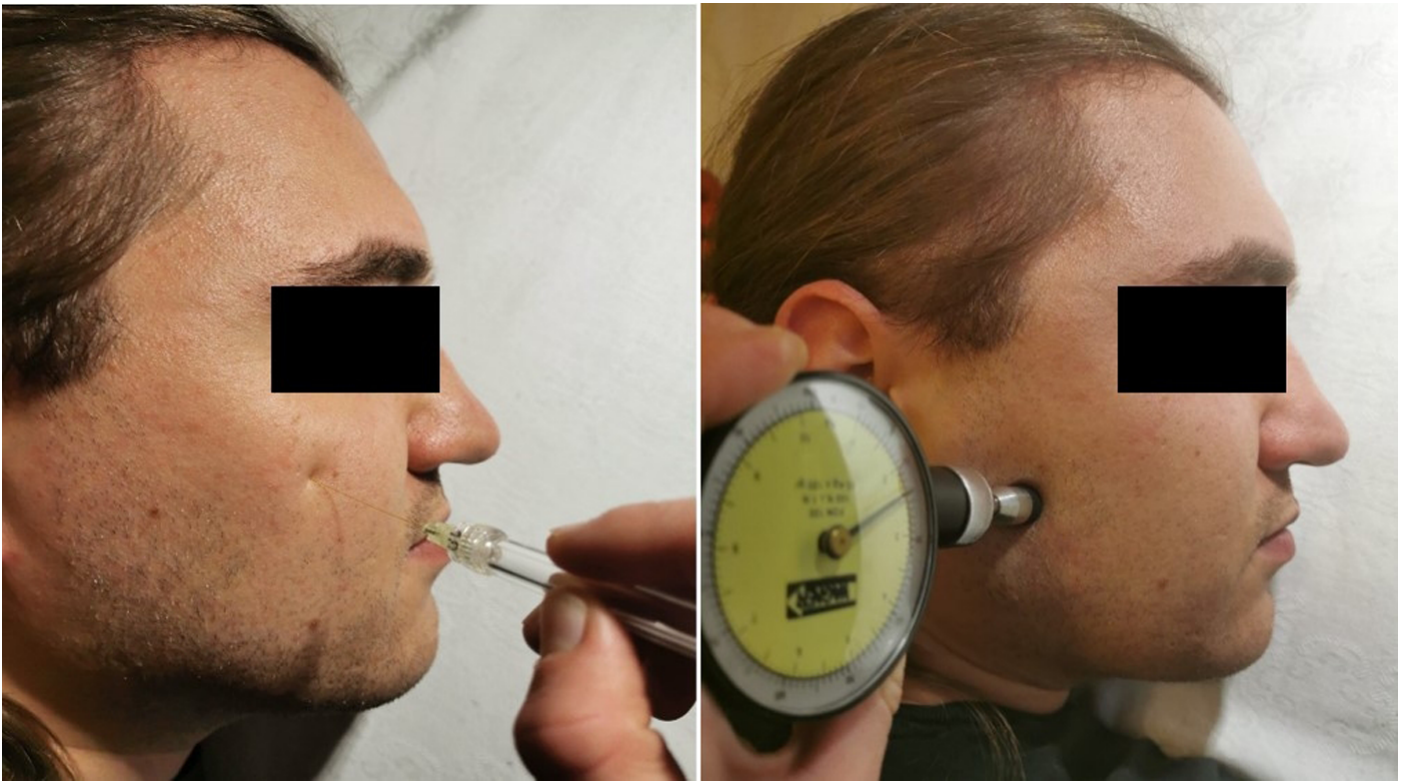
Left: Investigation of the mechanical detection threshold (MDT) at a standard spot on the face using von Frey hairs. Right: Investigation of the pressure pain threshold (PPT) using a pressure algometer on the face.

QST allows standardized testing of the somatosensory function of the face. The standardized QST protocol from the German Research Network on Neuropathic Pain (DFNS) includes the measurement of 13 parameters representing all fiber types and both peripheral and central nociception (13, 21) The following order was used: cold detection threshold (CDT), warm detection threshold (WDT), thermal sensory limen (TSL), paradoxical heat sensation (PHS), cold pain threshold (CPT), heat pain threshold (HPT), mechanical detection threshold (MDT), mechanical pain threshold (MPT), mechanical pain sensitivity (MPS), dynamic mechanical allodynia (DMA), wind-up ratio (WUR), vibration detection threshold (VDT), and pressure pain threshold (PPT). The examination method and equipment for QST standardized by the DFNS for measuring sensory profiles were used (13, 23). A thermal sensory analyzer (TSA-II; Medoc, Ramat Yishai, Israel) was used for the thermal testing (CDT, WDT, TSL, PHS, CPT, HPT). For MDT, von Frey filaments (von Frey hairs Optihair2-Set; Marstock Nervtest, Schriesheim, Germany) were used. For MPT, a set of seven weighted pinprick stimulators (MRI Compatible Pinprick Stimulator Set 8-512 mN; MRC Systems GmbH, Heidelberg, Germany) was used. For MPS, DMA, and WUR, the same seven weighted pinprick stimulators combined with a set of three tactile stimulators (SenseLab Brush-05; Somedic, Sweden; Q-tip fixed on an elastic strip and a cotton wisp) were applied. A tuning fork with cushioning (128 Hz; MedPlus, Radeberg, Germany) was used for the VDT. A pressure algometer (FDN100 plus rubber tip, 1 cm^2^; Algometer; Wagner Instruments, Greenwich, CT, United States) was used for measuring PPT. All tests were first performed on the back of a preferred hand, depending on the result of the Edinburgh Handedness Inventory. The hand is a standard area for QST assessments and was chosen as a control to rule out a sensory dysfunction independent from the facial palsy. A hairless 1–2 cm^2^ area between the thumb and forefinger was chosen. Next, the contralateral hemiface and then the paretic hemiface were tested. On the face, a 1–2 cm^2^ area below the zygomatic bone and approximately 4 cm horizontally distant from each nostril was selected for examination (Figure 1). For the VDT, the styloid process of the ulna was used on the hand, and the zygomatic process in the face was chosen. The complete test battery took 90 min per patient.

### Statistics

Statistical analyses were performed using IBM SPSS version 25.0 statistical software for Windows (Chicago, IL, United States). The evaluation strictly followed the protocol of the DFNS (12, 14). All QST parameters except CPT and HPT were logarithmically transformed to obtain a normal distribution. In the next step, a z-transformation was performed using “eQuiSTA” software (Casquar GmbH, Bochum, Germany). The *z*-values enable a comparison of the data independent of age, region, and gender using the DFNS reference database (13, 15, 16, 23). Z-scores of 0 correspond to the mean value of the healthy control group. Absolute sensory abnormalities for each parameter were defined as *z*-scores outside the 95% confidence interval (CI) of the reference group (*z*-score <−1.96 or >1.96). Values >0 indicate an increase in sensitivity (hyperesthesia), and values <0 indicate a loss of sensitivity (hypoesthesia). DMA and PHS are not normally present in healthy subjects, thus *z*-transformation was not performed. The *z*-scores (or raw data for DMA and PHS) of the paretic side and the contralateral side were compared using a paired *t*-test. The *z*-scores (or raw data for DMA and PHS) of patients with acute vs. chronic palsy were compared using a *t*-test. McNemar's test was used to compare the frequency of sensory abnormalities between the paretic and contralateral sides. Spearman's correlation was used to correlate the HB facial nerve grading, the FaCE scores, or the SF-36 scores with the *z*-scores of the QST parameters. *p* values were reported as two-tailed with a significance level of 5%.

## Results

### Demographics and questionnaires

Twenty-nine patients (22 female, median age of 48 years) were investigated. The etiology was idiopathic in 12 cases, traumatic in two cases, six cases had herpes zoster, and nine cases developed a postoperative palsy after benign tumor (parotid tumor, vestibular schwannoma) surgery. Seven patients had an acute facial palsy (range, 1–10 days after onset). Twenty-two patients had a chronic facial palsy (3.1–118 months after onset, 11 patients, chronic flaccid palsy; 11 patients, postparalytic syndrome with synkinesis). Table 1 summarizes the mimic facial function of the patients. The HB grading varied from grade II to grade VI. The average FaCE total score showed a relevant dysfunction (59.1 ± 15.5). Additionally, the FaCE facial discomfort subscale showed, on average, a relevant dysfunction (55.2 ± 28.2). Fifteen patients had a SF-36 pain subdomain score of 100, i.e., no facial pain, and seven patients had a pain score of <70 (average score, 85.1 ± 19.0). The average SF-36 general health subdomain score was 60.7 ± 17.8.

**Table 1:** Patients’ characteristics.

No.	Gender	Age (years)	FP	HB	FaCE Fcom	FaCE total	SF-36 Pain	SF-36 GH
1	male	25	chronic	II	100.0	76.67	100	67
2	female	48	chronic	IV	33.3	43.33	100	92
3	female	70	chronic	VI	50.0	46.67	100	87
4	female	41	chronic	II	33.3	48.30	100	77
5	female	60	chronic	II	91.7	60.00	51	40
6	female	58	chronic	III	91.7	66.67	100	40
7	female	45	chronic	III	8.3	46.67	62	52
8	female	55	chronic	IV	50.0	51.67	100	52
9	female	54	chronic	IV	41.7	33.33	100	57
10	female	67	chronic	IV	33.3	50.00	62	57
11	female	46	chronic	II	66.7	61.67	100	97
12	female	65	chronic	III	41.7	61.67	74	62
13	female	34	chronic	III	41.7	60.00	62	62
14	female	27	chronic	II	16.7	60.00	100	67
15	female	33	chronic	III	50.0	70.00	100	77
16	female	73	chronic	III	83.3	60.00	84	57
17	female	68	chronic	III	33.3	62.50	42	40
18	female	64	chronic	II	100.0	64.29	80	44
19	female	40	chronic	II	58.3	68.33	100	82
20	female	56	chronic	IV	50.0	48.33	84	77
21	female	71	chronic	II	33.3	60.00	100	67
22	female	32	chronic	II	25.0	36.67	62	62
23	male	36	acute	IV	50.0	63.33	100	52
24	male	18	acute	IV	33.3	45.00	72	30
25	male	63	acute	II	50.0	46.67	100	40
26	male	21	acute	II	100.0	85.00	100	72
27	male	43	acute	III	33.3	45.00	80	47
28	male	26	acute	III	100.0	100.00	100	32
29	female	61	acute	III	100.0	91.67	52	72
Mean		48.1			55.2	59.1	85.1	60.7
SD		16.8			28.2	15.5	19.0	17.8
Median		48			50	60	100	62.0

FP, facial palsy; HB, house-brackmann scale; FaCE, facial clinimetric evaluation scale; Fcom, facial comfort subscale; SF-36, 36-item short form survey; Pain, pain subdomain; GH, general health subdomain, SD, standard deviation.

### Quantitative sensory testing on the paretic and contralateral hemifaces

**Figure 2:**
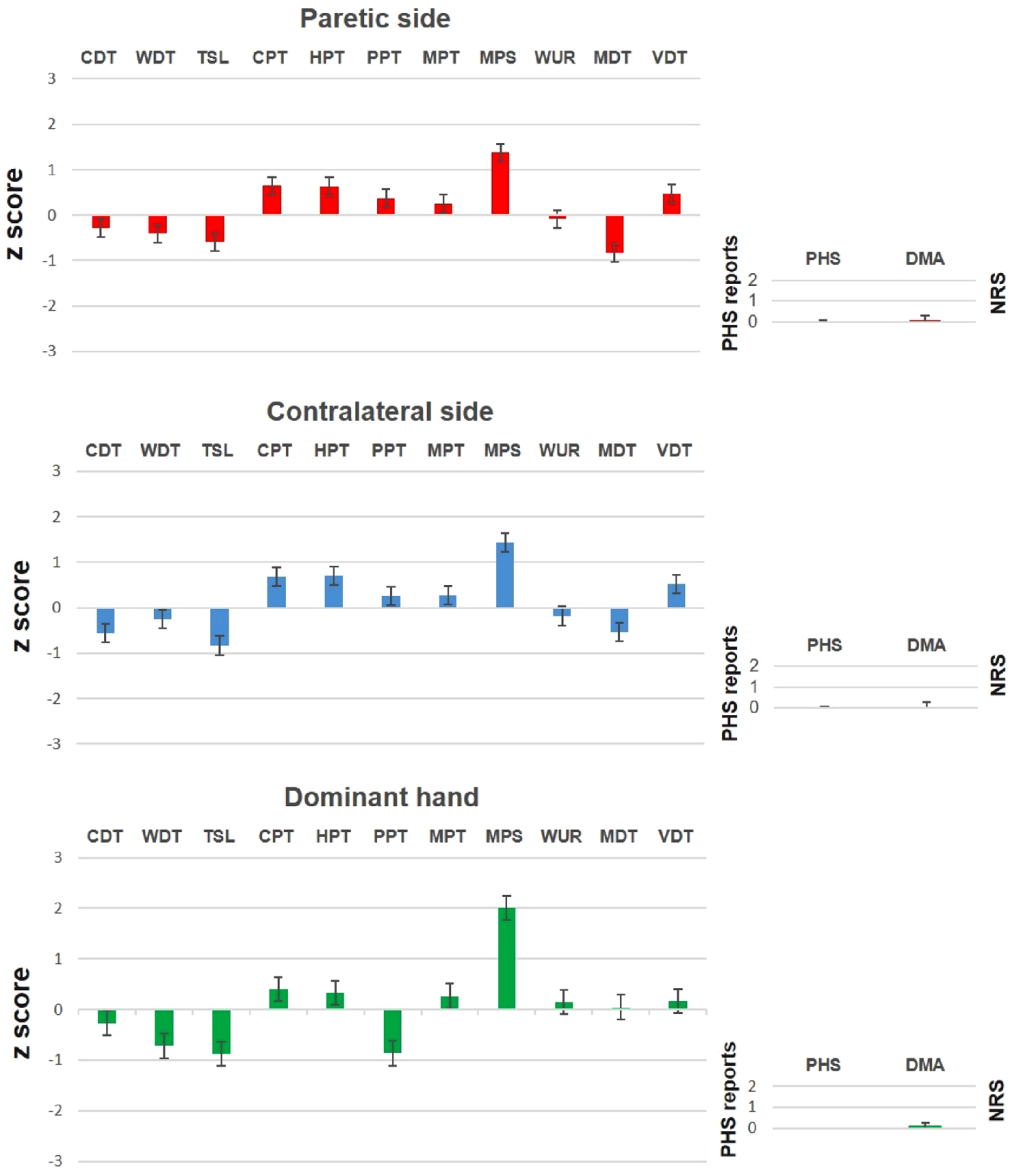
The somatosensory *z*-profiles of the paretic side (upper row), the contralateral side (middle row), and the dominant hand (lower row) in patients with facial palsy compared with controls. PHS and DMA were not *z*-transformed and are presented as raw data. Data are mean ± standard error. CDT, cold detection threshold; WDT, warm detection threshold; TSL, thermal sensory limen; CPT, cold pain threshold; HPT, heat pain threshold; PPT, pressure pain threshold; MPT, mechanical pain threshold; MPS, mechanical pain sensitivity; WUR, wind-up ratio; MDT, mechanical detection threshold; VDT, vibration detection threshold; PHS, paradoxical heat sensation; DMA, dynamical mechanical allodynia.

The frequencies of QST abnormalities on both sides of the face are shown in Table 2. Many but not all patients had normal somatosensory function in most subtests. WUR, VDT, and PHS was normal for all 29 patients on both sides of the face. Twenty-five patients had normal PPT, DMA, MPT, CPT, TSL, and WDT. MPS was normal for 20 patients on the paretic side and for 21 patients on the contralateral side. Only the MDT score showed a large group of patients with abnormal values on both sides of the face. Here, only 13 patients had a normal score on the paretic side and 16 patients had a normal score on the contralateral side. Both types of abnormality were seen, i.e., hyperesthesia or hypoesthesia, without preponderance for most tests. If the MPS score was abnormal, the patients always had hyperesthesia in the face. On average, the frequencies of abnormalities were not different between the paretic and contralateral sides for any of the parameters (all *p* > 0.05). Additionally, when compared with healthy controls from the DFNS reference database using the *z*-scores (Table 3 and Figure 2), there were no differences between the paretic and contralateral sides (all *p* > 0.05). Furthermore, there were no differences between patients with acute facial palsy and those with chronic facial palsy on the paretic and contralateral sides (all *p* > 0.05). The total FaCE score, the FaCE facial comfort score, the SF-36 pain subdomain score, and the SF-36 general health subdomain score did not correlate with the QST parameters (all *p* > 0.05). A higher HB grading (worse facial function) was correlated with a lower CDT normalized score (more hypoesthesia) on both sides of the face (paretic side, *ρ* = −0.416; *p* = 0.025; contralateral side, *ρ* = −0.391; *p* = 0.036). Such correlations were not seen on the paretic or contralateral sides for other QST parameters (all *p* > 0.05).

**Table 2:** Absolute number of hemifaces with increased (hyperesthesia), normal, and decreased sensory function (hypoesthesia)^a^.

Parameter	Hyperesthesia *N*	Normal *N*	Hypoesthesia *N*	*p*
CDT Paretic side	0	28	1	0.352
CDT Contralateral side	0	28	4	
WDT Paretic side	0	26	3	1.000
WDT Contralateral side	0	27	2	
TSL Paretic side	0	27	2	1.000
TSL Contralateral side	0	26	3	
CPT Paretic side	3	26	0	1.000
CPT Contralateral side	2	27	0	
HPT Paretic side	3	25	1	0.465
HPT Contralateral side	4	25	0	
PPT Paretic side	1	28	0	1.000
PPT Contralateral side	1	28	0	
MPT Paretic side	1	27	1	0.355
MPT Contralateral side	0	29	0	
MPS Paretic side	9	20	0	1.000
MPS Contralateral side	8	21	0	
WUR Paretic side	0	29	0	NA
WUR Contralateral side	0	29	0	
MDT Paretic side	7	13	9	0.704
MDT Contralateral side	5	16	8	
VDT Paretic side	0	29	0	NA
VDT Contralateral side	0	29	0	
PHS Paretic side	0	29	0	NA
PHS Contralateral side	0	29	0	
DMA Paretic side	0	27	2	1.000
DMA Contralateral side	0	26	3	

CDT, cold detection threshold; WDT, warm detection threshold; TSL, thermal sensory limen; CPT, cold pain threshold; HPT, heat pain threshold; PPT, pressure pain threshold; MPT, mechanical pain threshold; MPS, mechanical pain sensitivity; WUR, wind-up ratio; MDT, mechanical detection threshold; VDT, vibration detection threshold; PHS, paradoxical heat sensation; DMA, dynamical mechanical allodynia.

^a^ Raw data were transformed to *z*-scores. Scores within the 95% confidence interval (CI) of the reference group (*z*-score <1.96 or >1.96) are defined as normal. Values >1.96 indicate an increase in sensitivity (hyperesthesia), and values <−1.96 indicate a loss of sensitivity (hypoesthesia).

**Table 3:** Comparison of the *z*-scores of the differences between the paretic and contralateral sides.

Parameter	Mean	Standard deviation	Lower 95% CI	Upper 95% CI	*p*
CDT Paretic/Contralateral side	−0.063	0.293	−0.175	0.048	0.253
WDT Paretic/Contralateral side	0.031	0.338	−0.098	0.160	0.625
TSL Paretic/Contralateral side	−0.063	0.190	−0.136	0.009	0.083
CPT Paretic/Contralateral side	−0.326	4.737	−2.127	1.476	0.714
HPT Paretic/Contralateral side	0.254	2.688	−0.768	1.277	0.615
PPT Paretic/Contralateral side	−0.011	0.095	−0.047	0.026	0.550
MPT Paretic/Contralateral side	0.016	0.415	−0.145	0.177	0.836
MPS Paretic/Contralateral side	−0.026	0.122	−0.073	0.020	0.258
WUR Paretic/Contralateral side	0.022	0.125	−0.025	0.070	0.344
MDT Paretic/Contralateral side	0.097	0.442	−0.071	0.265	0.249
PHS Paretic/Contralateral side^a^	NA	NA	NA	NA	NA
VDT Paretic/Contralateral side	−0.047	0.453	−0.219	0.126	0.585
DMA Paretic/Contralateral side	−0.002	0.080	−0.032	0.028	0.890

CDT, cold detection threshold; WDT, warm detection threshold; TSL, thermal sensory limen; CPT, cold pain threshold; HPT, heat pain threshold; PPT, pressure pain threshold; MPT, mechanical pain threshold; MPS, mechanical pain sensitivity; WUR, wind-up ratio; MDT, mechanical detection threshold; VDT, vibration detection threshold; DMA, dynamical mechanical allodynia.

^a^ PHS, no patient developed a paradoxical heat sensation; NA, not applicable.

### Quantitative sensory testing on the dominant hand as a control area

The *z*-scores and the frequencies of QST abnormalities on the dominant hand are listed in Supplementary Table S1. On average, the group of patients did not show abnormalities. What was striking was that only hyperesthesia was evident (increased *z*-score) with the mechanical pain sensitivity test in the dominant hand of nearly half of the participants.

## Discussion

QST includes both thermal and mechanical test stimuli, allows a complete somatosensory profiling of one affected area and unaffected control areas, and was applied for the first time for the faces of patients with unilateral facial palsy. Overall, the study population did not show abnormalities on the dominant hand and on both sides of the face, i.e., they had normal sensory function in the face, as shown by QST. In one or two subtests, a few patients had a pathological value, but without predominance on the side of the facial palsy. Furthermore, some patients had pathological results from the predominant hand (either hyperesthesia or hypoesthesia) that did not correlate with the results from the face. We do not have a clear explanation for these pathological results in the contralateral face or on the predominant hand. By contrast to the overall unremarkable results in patients with facial palsy, QST has been proven to be a reliable tool for detecting sensory dysfunction in the face; for instance, for patients with trigeminal neuralgia or numerous other diseases, or for the application of local anesthesia in the face (24–26).

Domains of the FaCE and the SF-36 were used to investigate the feeling of numbness with validated PROMs. Only five patients had a normal FaCE facial comfort score (value of 100). Furthermore, 13 patients had pain in the SF-36 pain subdomain (value <100). The SF-36 does not register the localization of the pain, and we did not ask these patients with abnormal SF-36 pain scores for a localization. In addition, whether the FaCE facial comfort score or any subdomain of the SF-36 exactly address this felt numbness. This might explain why the PROM values did not correlate with the QST results. The patients typically feel the numbness in the entire hemiface. The feeling of numbness is not restricted to the dermatome distribution of the trigeminal nerve. Nevertheless, it is unlikely that any sensory abnormality is restricted to only a part of the paralyzed hemiface. This fits with a recent study analyzing six areas of the faces of patients with Bell's palsy that did not reveal any differences between the areas (27). We had to limit the assessment to one well-defined spot in the midface, mainly for practical reasons. The analysis of one spot took 30 min. Therefore, a broader mapping of the face is not realistic for a larger sample of probands. Nevertheless, the restriction of the assessment to a small area of the face, the small sample size, and the heterogeneity of etiologies were clear limitations of the study. Furthermore, the feeling of numbness is different from postauricular pain. The first occurs after the onset of the palsy and is not equal to pain, whereas the latter is a prodromal syndrome before the palsy occurs (28). It might be that the pain threshold is decreased in the retroauricular region in the acute phase of the disease (29). Hence, it might be of interest to perform a QST study in the ear region of the patients.

For most patients, the QST examination did not reveal hyperesthesia or a hypoethesia. Hyperesthesia occurred at the same frequency as hypoesthesia (in 1–9 patients in the QST subtests). When there are reports of clinical neurological examinations showing hypoesthesia in the paretic area, Adour et al. are usually cited (30); although the first report was published by Ch'ien and Halsey (31). It has to be noted that both studies did not report their testing method. May and Harden evaluated 500 patients with acute Bell's palsy; 10% had decreased pin prick, pin scratch, or light touch sensibility (32). Novak et al. examined facial sensibility in 29 patients with acute or chronic facial palsy (10). They used vibratory and cutaneous pressure thresholds and two-point discrimination measurements. They found abnormalities in approximately half of the patients and a non-significant statistical trend for higher thresholds on the affected side. From today's perspective, these studies do not fulfill the high standards for a reliable measurement of facial sensibility. In a recent study, Cárdenas Palacio et al. examined the facial sensibility with pressure threshold and two-point discrimination in six areas of the face in 12 patients with Bell's palsy over a 2- to 6-week period (three or four patients per timepoint) (27). Significant differences were observed between both sides of the face with a two-point discrimination test on eyelid, cheek, and lip. A comparison and standardization to normal values, as recommended by the DFNS, was not performed. Hence, the informative value of the study remains doubtful.

Some reports have postulated the occurrence of trigeminal nerve dysfunction in patients with facial palsy, based on trigeminus-evoked potential or blink reflex testing. In smaller series, approximately 30%–50% of the patients showed a pathological blink reflex related to the afferent (trigeminal) part (9, 11). It is important to note that, theoretically, these pathological results are probably related to a brainstem dysfunction and are not proof of peripheral trigeminal dysfunction. This means that the question as to why many patients with facial palsy feel numbness in the affected hemiface remains unanswered. The nervous separation of input (afferent: trigeminal nerve) and output (efferent: facial nerve) in the face is unique in mammals. The inability to execute a motor signal due to peripheral facial nerve lesion requires a central nervous system mechanism to handle the perceptual-motor mismatch (8). The role of the somatosensory feedback, particularly the sensory-motor interplay in the adaptation process, is poorly understood (8). A task-based and resting-state functional MRI study of 24 patients with acute Bell's palsy showed an involvement of the somatosensory system and the thalamus in the adaptation process (8). The increased connectivity between subcortical and cortical structures indicated an active sensory-motor adaptation process. The authors hypothesized that the decreased functional connectivity at the cortical level reflects an unsuccessful sensorimotor adaptation process due to an inability to solve the somatosensory-motor mismatch. Furthermore, the local sensorimotor network processing efficiency in patients with chronic facial palsy seems to be permanently reduced (33). The mismatch and the altered sensorimotor central nervous system processing might be felt as a “strange feeling” that should be elaborated in future studies correlating the cortical mismatch with a battery of sensibility testing in the face. Treatment strategies to overcome this mismatch should have an impact on recovery or the relief of facial palsy-related symptoms.

## Conclusions

Using QST to undertake a comprehensive and systematic analysis of facial sensitivity, the examination of 29 patients with unilateral acute or chronic facial palsy did not reveal a sensory dysfunction in the face. Sensory stimulation elements can be included in rehabilitation programs for patients with facial palsy and are expected to work for certain subsets of patients. Moreover, treatment concepts need to be developed to overcome the suspected cortical sensorimotor mismatch.

## Data Availability

The original contributions presented in the study are included in the article/**Supplementary Material**, further inquiries can be directed to the corresponding author.
