# Supplementary material for: Sensory function in the faces of patients with facial palsy: A prospective observational study using quantitative sensory testing
Source: Front Pain Res (Lausanne). 2022 Dec 19;3:1041905. doi: 10.3389/fpain.2022.1041905 (PMC9806347; doi:10.3389/fpain.2022.1041905)
Supplement: Supplementary file 1 [file Table1.docx]

**Supplement TABle 1**

**Sensory function in the face of patients with facial palsy: A prospective observational study using quantitative sensory testing**

Gerd Fabian Volk, MD^1,2,3^, Marianna Döhler^1^, Carsten M. Klinger, MD^4^, Thomas Weiss, PhD^5^ and Orlando Guntinas-Lichius, MD^1,2,3^*

^1^Department of Otorhinolaryngology, Jena University Hospital, Jena, Germany

^2^Facial-Nerve-Center Jena, Jena University Hospital, Jena, Germany

^3^Center for Rare Diseases, Jena University Hospital, Jena, Germany

^4^Department of Neurology, Jena University Hospital, Jena, Germany

^5^Department of Psychology, Clinical Psychology, Friedrich Schiller University, Jena, Germany

| **Supplement Table 1.** | | | | | |
| --- | --- | --- | --- | --- | --- |
| **Parameter** | **Mean** | **SE** | **Hyperesthesia**  **N** | **Normal**  **N** | **Hypoesthesia**  **N** |
| CDT Dominant hand | -0.27 | 0.17 | 0 | 27 | 2 |
| WDT Dominant hand | -0.71 | 0.26 | 0 | 24 | 5 |
| TSL Dominant hand | -0.88 | 0.18 | 0 | 24 | 5 |
| CPT Dominant hand | 0.40 | 0.17 | 2 | 27 | 0 |
| HPT Dominant hand | 0.33 | 0.20 | 2 | 27 | 0 |
| PPT Dominant hand | -0.86 | 0.25 | 0 | 23 | 6 |
| MPT Dominant hand | 0.26 | 0.13 | 0 | 29 | 0 |
| MPS Dominant hand | 2.01 | 0.16 | 13 | 16 | 0 |
| WUR Dominant hand | 0.15 | 0.16 | 0 | 29 | 0 |
| MDT Dominant hand | 0.04 | 0.14 | 0 | 29 | 0 |
| VDT Dominant hand | 0.17 | 0.14 | 0 | 29 | 0 |
| PHS Dominant hand | -0.15 | 0.00 | 0 | 29 | 0 |
| DMA Dominant hand | 1.25 | 3.71 | 4 | 25 | 0 |

*Raw data were transformed to z scores. Scores within the 95% confidence interval (CI) of the reference group (z-score < 1.96 or > 1.96) are defined as normal. Values > 1.96 indicate an increase in sensitivity (hyperesthesia), values < -1.96 indicate a loss of sensitivity (hypoesthesia). CDT= cold detection threshold, WDT= warm detection threshold, TSL= thermal sensory limen, CPT= cold pain threshold, HPT= heat pain threshold, PPT= pressure pain threshold, MPT= mechanical pain threshold, MPS= mechanical pain sensitivity, WUR= wind-up ratio, MDT= mechanical detection threshold, VDT= vibration detection threshold, PHS= paradoxical heat sensation, DMA= dynamical mechanical allodynia
